# Dual function of *Candida auris* mannosyltransferase, MNT5, in biofilm community protection from antifungal therapy and the host

**DOI:** 10.1128/mbio.00346-25

**Published:** 2025-02-25

**Authors:** Robert Zarnowski, Mark V. Horton, Chad J. Johnson, Por Choua Vang, Jeremy Uram, Liyanage Devthilini Pasasum Fernando, Jiri Vlach, Christian Heiss, Parastoo Azadi, Jeniel E. Nett, David R. Andes

**Affiliations:** 1 Department of Medicine, University of Wisconsin-Madison, Madison, Wisconsin, USA; 2 Department of Medical Microbiology and Immunology, University of Wisconsin-Madison, Madison, Wisconsin, USA; 3 Complex Carbohydrate Research Center, University of Georgia, Athens, Georgia, USA; Duke University School of Medicine, Durham, North Carolina, USA

**Keywords:** *Candida auris*, biofilms, drug resistance mechanisms, host response, cell wall, extracellular matrix

## Abstract

Screen of mutants from a mannosyltransferase family identified the importance of *MNT5* for *C. auris* biofilm drug resistance and neutrophil evasion. Biochemical analysis of the *mnt5∆* mutant matrix and cell wall identified alterations in the mannan structures. Resistance and matrix for *mnt5∆* were restored with delivery of wild-type matrix via extracellular vesicles. Analysis of the *mnt5*∆ cell wall revealed a reduction in mannan and compensatory increase in cell surface glucan and chitin, suggesting a role for *MNT5* in mannan masking of pathogen-associated molecular patterns.

**IMPORTANCE:**

*C. auris* recalcitrance is linked to biofilm drug resistance and immune evasion. The mannosyltransferase encoded by *MNT5* is necessary for both phenotypes and may serve as a useful therapeutic target.

## OBSERVATION

*Candida auris* is an emerging fungal species responsible for healthcare-associated outbreaks and infections associated with relatively poor patient outcomes (1–6). The clinical features of these infections include biofilm persistence on abiotic surfaces and skin, immune evasion, and antifungal drug resistance (7–12). Many of the mechanisms underlying these distinct phenotypes remain largely unknown.

Cell wall and extracellular matrix polysaccharides have mechanistic roles implicated in the protection of *Candida* species from host response and drug therapies (13–16). In the biofilm community, a unique extracellular mannan-glucan complex has been shown to prevent antifungal drugs from reaching intracellular drug targets (17–20). Similarly, cell wall mannan masks immunostimulatory cell wall components preventing host cell recognition (15, 21). The present investigations explore the function and genetic control of mannosylation in the biofilm and host scenarios for *C. auris* via screen of a family of six mannosyltransferase mutants encoded by *MNT1*, *MNT2*, *MNT3*, *MNT4*, *MNT4b*, and *MNT5* (Table S1). We sought to test the hypothesis that *C. auris* mannan production is important for (i) matrix-associated antifungal drug resistance and (ii) protection from the host.

### *C. auris MNT5* impacts biofilm drug resistance

**Fig 1:**
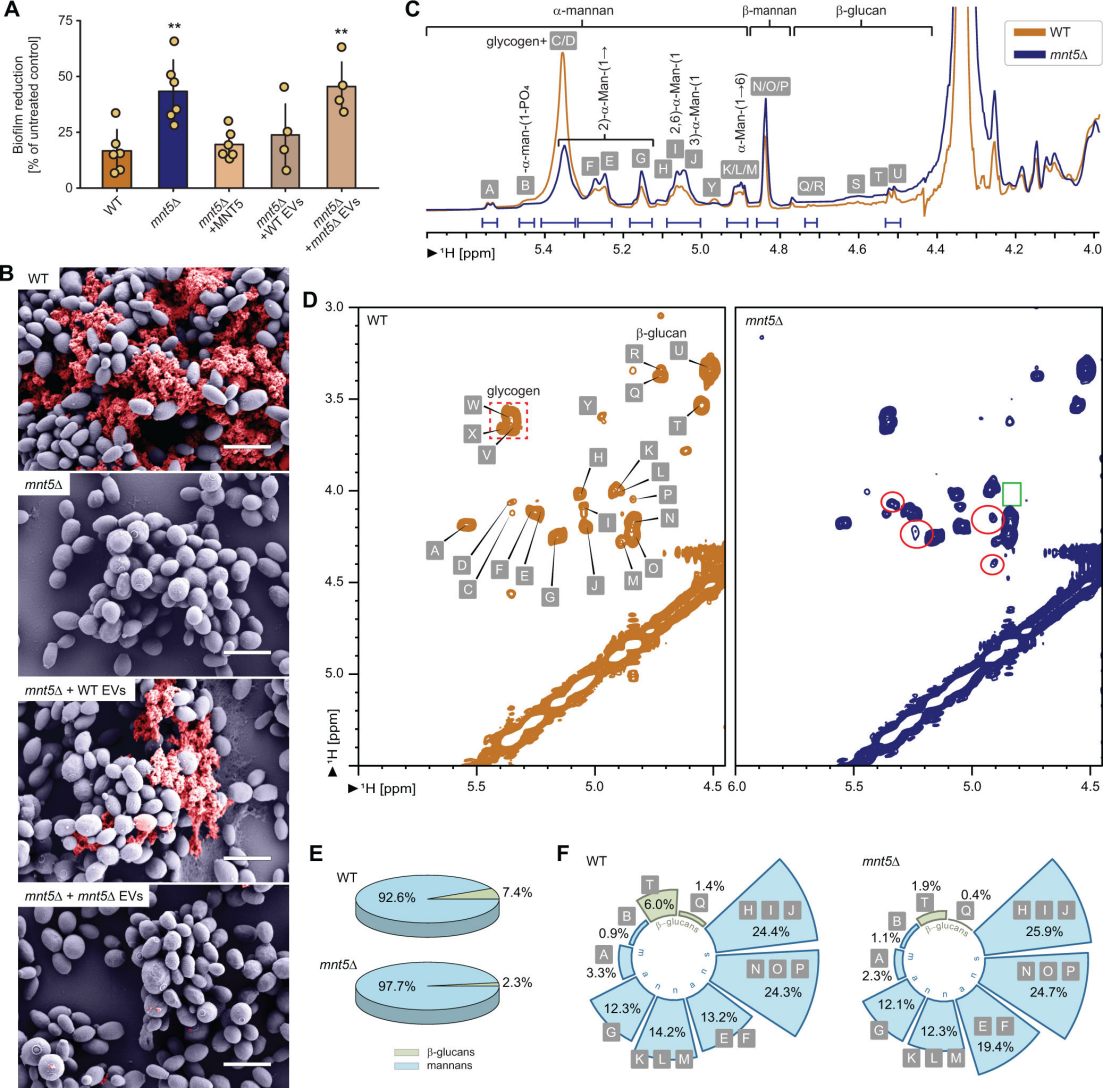
Abnormalities in the extracellular matrix of *C. auris mnt5*∆ mutants impact biofilm antifungal susceptibility. (**A**) The percent reduction in biofilm formation was measured following treatment with fluconazole (1000  µg/mL), compared to untreated biofilms. The *mnt5*∆ mutant exhibited an enhanced susceptibility phenotype, whereas the complementation strain restored this phenotype to the wild-type level. A return toward the reference *C. auris* wild-type resistant phenotype was observed following exogenous wild-type EV addition to the mutant biofilm. Addition of EVs from the *mnt5*∆ strain did not alter the *mnt5*∆ susceptible phenotype. Each dot represents an independent biological replicate and reflects the mean of eight technical replicates. Error bars denote standard deviation. A nonparametric Kruskal–Wallis one-way analysis of variance with an uncorrected Dunn’s multiple comparison test was performed, with a significant *P* value indicated as <0.0001. (**B**) The biofilm matrix surrounding *C. auris* biofilm from the wild-type and *mnt5*∆ mutant strains in 24 hour old biofilms grown on *in vitro* coverslips was visualized using scanning electron microscopy. A return toward the reference *C. auris* wild-type matrix was observed following exogenous wild-type EV addition to the mutant biofilm. Addition of EVs from the *mnt5*∆ strain did not restore the visual matrix phenotype. Biofilm fungal cells were pseudocolored in grey-blue, while the extracellular matrix is shown in brick red. Scale bar  =  2  µm. (**C**) An overlay of 1D ^1^H NMR spectra highlighting the anomeric region of the detected glycans in the extracellular matrices of *C. auris* biofilms from the wild-type and *mnt5*∆ mutant strains. Resonances between 5.5 and 4.9 ppm were attributed to the anomeric protons of α- and β-mannans, while signals in the 4.0–4.7 ppm range corresponded to β-glucans, specifically β-(1→6)-linked glucan side chains (4.51 ppm) and β-(1→3)-linked glucan backbones (4.72 ppm). Comparative analysis revealed a similar overall carbohydrate composition but differences in the concentrations of glycan components between the two strains. (**D**) Comparison of 2D ^1^H-^1^H COSY spectra of *C. auris* wild-type and *mnt5*∆ mutant biofilm extracellular matrices revealed the emergence of new peaks and the disappearance or intensity reduction of others in the *mnt5*∆ mutant, particularly within the α- and β-mannan regions. Notably, the *mnt5*∆ mutant exhibited new peaks at 5.24/4.22 ppm, tentatively assigned to →2)-α-Man-(1→3). Additional new peaks were observed at 4.91/4.15 ppm and 4.90/4.39 ppm, likely corresponding to →2)-β-Man-(1→. A peak at 4.84/4.05 ppm, corresponding to β-Man in the β-Man-(1→2)-α-Man-(1→PO4 fragment, was significantly reduced in the *mnt5*∆ mutant. Identified peaks are labeled in the wild-type strain spectrum, with new peaks circled in red and the position of a missing peak highlighted in a green box. The peaks labeled W, V, X, and Y corresponded to glucosyl residues connected by α-(1→4)- and α-(1→6)-glycosidic bonds. (**E**) α- and β-mannans are dominant components of the extracellular matrices of *C. auris* wild-type and *mnt5*∆ mutant biofilms. The proportion of mannan residues was slightly increased in the *mnt5*∆ mutant (97.7%) compared to the wild-type (92.6%), while the β-glucan content decreased in the *mnt5*∆ mutant (2.3%) compared to the wild-type (7.4%). (**F**) Relative abundance of individual glycosidic residues identified in *C. auris* wild-type and *mnt5*∆ mutant biofilm extracellular matrices, based on the integration of the anomeric peaks in the 1D ^1^H NMR spectra.

We constructed *C. auris* mutants with disruption of *MNT* genes and assessed their biofilm growth and antifungal susceptibility to fluconazole during biofilm formation (Fig. 1A; Fig S1). Growth defects were not noted (not shown). We found the biofilms of the *mnt5*∆ mutant to exhibit increased drug susceptibility during biofilm growth. Compared to the wild-type, antifungal treatment of the *mnt5*∆ biofilm resulted in more than double the fungal killing (Fig. 1A). The increased drug susceptibility was reversed by genetic complementation, thus validating the mutants’ genotype-phenotype relationship. *MNT5* disruption did not alter planktonic minimum inhibitory concentrations to fluconazole, suggesting that this *MNT5* function is specific to the biofilm state (not shown).

### *C. auris MNT5* impacts functional biofilm matrix production

Previous studies have demonstrated the importance of the extracellular matrix in protection of the *Candida* biofilm community from antifungal drugs, including for *C. auris* (17, 18, 22). Given the enhanced fluconazole susceptibility of the *mnt5*∆ mutant, we reasoned that the mutant may exhibit either a quantitative or qualitative alteration in the biofilm matrix. We first analyzed the matrix via scanning electron microscopy of biofilms grown on coverslips *in vitro*. Imaging of the *mnt5*∆ mutant biofilms revealed a marked reduction in the community encasing extracellular matrix material (Fig. 1B). Nuclear magnetic resonance (NMR) analysis demonstrated that the extracellular matrix of the wild-type and *mnt5*∆ mutant contained similar monosugar composition but exhibited differences in the abundance of α- mannan, β-mannan, β-(1→6)-glucan, and β-(1→3)-glucan linkages (Fig. 1C; Table S7). Spectral analysis of the *mnt5*∆ matrix revealed new peaks assigned to →2)-α-Man-(1→3) and →2)-β-Man-(1→, whereas a peak corresponding to β-Man in the β-Man-(1→2)-α-Man-(1→PO4 fragment was highly reduced in the mutant (Fig. 1D). This finding is consistent with altered α-mannan structures and new moieties in the biofilm extracellular matrix of *mnt5*∆ mutant. The proportion of mannan residues was slightly increased in the *mnt5*∆ mutant (97.7%) compared to the wild-type (92.6%), while the β-glucan content decreased in the *mnt5*∆ mutant (2.3%) compared to the wild-type (7.4%) (Fig. 1E and F).

We previously found that matrix polysaccharides are delivered via extracellular vesicles (EVs) across the *Candida* genus. We theorized that *MNT5* governs matrix mannan deposition via EVs and that functional EVs from wild-type *C. auris* would reverse the *mnt5*∆ drug susceptibility phenotype. To test this, we added exogenous wild-type and *mnt5*∆ EVs to the *mnt5*∆ and analyzed drug resistance and the biofilm matrix accumulation. We observed a return of the *mnt5*∆ mutant susceptibility phenotype toward the wild-type *C. auris*-resistant phenotype. We similarly observed a return in the biofilm extracellular matrix with wild-type EVs. Conversely, the addition of EVs from the *mnt5*∆ did not restore these reference biofilm phenotypes. This suggests that *MTN5* is important for delivery of functional biofilm matrix in an EV-dependent manner (Fig. 1A and B).

### Genetic disruption of *C. auris* mannosylation enhances neutrophil engagement

**Fig 2:**
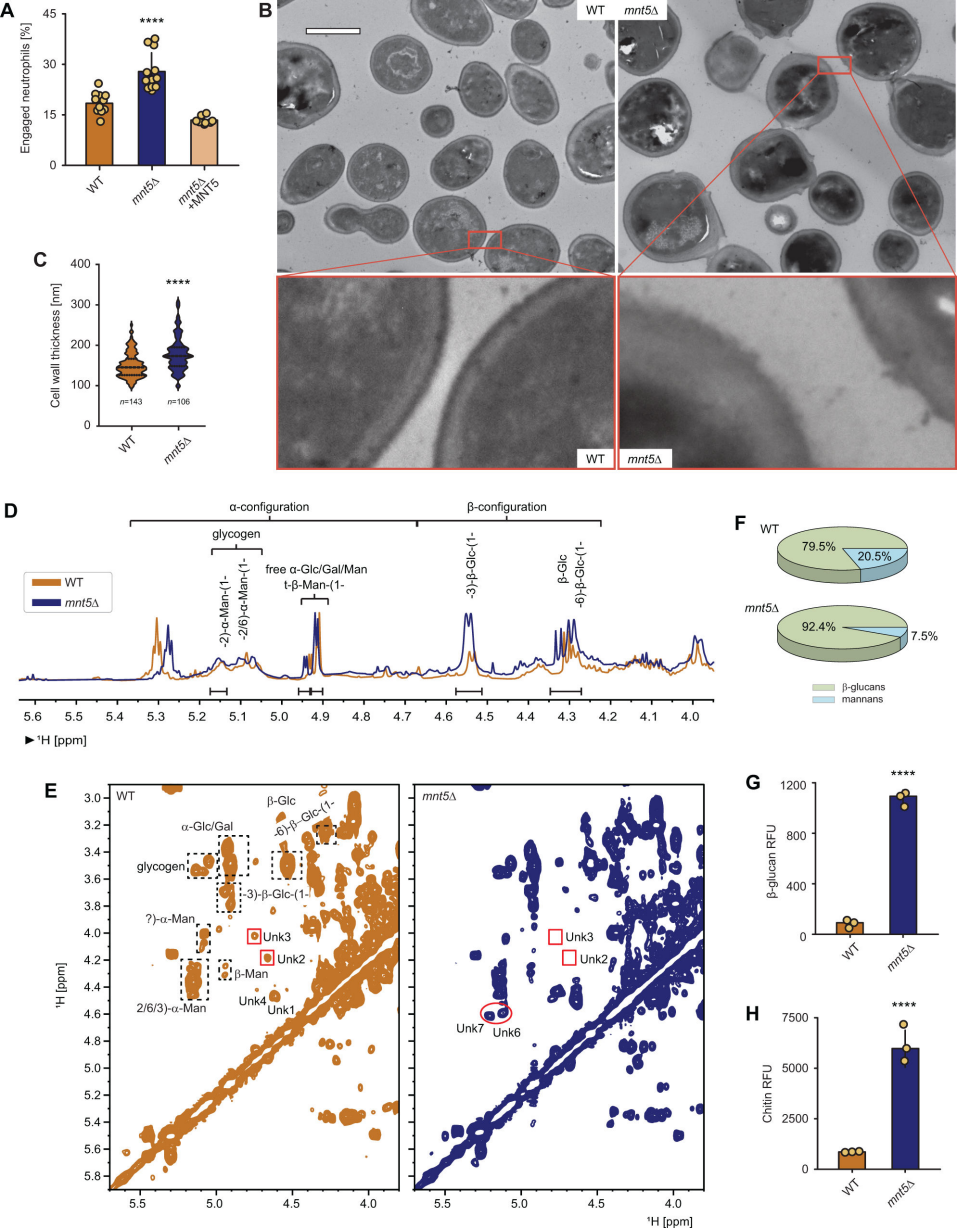
Genetic disruption of *C. auris MNT5* alters the cell wall mannan structure increasing PAMP exposure and neutrophil attack. (**A**) The *mnt5*∆ mutant cells are more prone to being engulfed by live neutrophils compared to the wild-type strain. Three independent biological replicates, each with 12 technical replicates (cell events), were performed. Error bars represent standard deviation. A nonparametric Kruskal–Wallis one-way analysis of variance with an uncorrected Dunn’s multiple comparison test was used, with a significant *P* value indicated as <0.0001. (**B**) Transmission electron microscopy (TEM) analysis of *C. auris* wild-type and *mnt5*∆ mutant cells revealed changes in the cell wall of the latter. Imaging was performed at 8,800× magnification, with a scale bar of 2 µm. High magnification images (66,000× magnification) show differences in overall cell wall thickness and electron density between the inner β-glucan and outer mannan layers. In the *mnt5*∆ mutant, the peripheral glycans appeared wider and less dense. (**C**) Quantification of cell wall thickness based on TEM images was performed manually using Fiji ImageJ. Ten measurements per cell were taken from a total of 143 wild-type cells and 106 MNT5 null mutant cells. Error bars represent standard deviation. A two-tailed unpaired *t*-test was applied, with a significant *P* value indicated as <0.0001. (**D**) An overlay of 1D ^1^H NMR spectra of *C. auris* wild-type and *mnt5*∆ mutant cell wall samples showed general similarities in the positions and multiplicities of carbohydrate peaks but revealed distinct differences in signal intensities between the profiles. The signal intensity variations observed in the *mnt5*∆ mutant, compared to the wild-type, indicate a relative increase in β-glucan content and a corresponding relative decrease in the mannan pool. In addition, differences in peak shapes were noted in the α-mannan region (5.05–5.2 ppm). (**E**) Comparison of 2D ^1^H-^1^H COSY spectra revealed variations between *C. auris* wild-type and *mnt5*∆ mutant cell walls. The Unk2 and Unk3 signals were absent in the *mnt5*∆ mutant (shown in red squares), while the Unk1 and Unk4 peaks became more intense. The 2D COSY spectra confirmed differences in peak shapes in the α-mannan region, with lower intensity peaks in the *mnt5*∆ mutant compared to the wild-type. Additionally, new peaks (Unk6 and Unk7, shown in red circles) emerged in the α-mannan region of the *mnt5*∆ mutant, suggesting structural alterations. Overall, the mannan content appeared lower in the *mnt5*∆ mutant compared to the wild-type. (**F**) The proportion of glucan was significantly higher in the *mnt5*∆ mutant (92.4%) compared to the wild-type (79.5%), while the mannan content was lower in the *mnt5*∆ mutant (7.5%) compared to the wild-type (20.5%). (**G**) *C. auris mnt5*∆ mutant displays increased amounts of β-glucan on the cell surface. The cell surface was labeled using Fc:dectin-1 protein with an Alexa Fluor 488-conjugated anti-human IgG Fc antibody and imaged by fluorescence microscopy. Error bars represent the standard deviation of three independent biological replicates. A two-tailed unpaired *t*-test was performed, with a significant *P* value indicated as <0.0001. (**H**) Surface presentation of chitin is increased in the *C. auris mnt5*∆ mutant compared to the wild-type strain. Cell surface chitin was labeled with wheat germ agglutinin conjugated to fluorescein isothiocyanate (WGA-FITC) and assessed by fluorescence microscopy. Error bars represent standard deviation. A two-tailed unpaired *t*-test was performed, with a significant *P* value indicated as <0.0001.

To explore the influence of the MNT mannosyltransferase family on neutrophil evasion for *C. auris*, we examined human neutrophil interactions with the strains during co-culture. Neutrophils engaged the *mnt5*∆ mutant at a greater than threefold higher rate than the wild-type strain (Fig. 2A; Fig S2). Genetic complementation of *mnt5*∆ mutant returned the host cell interaction toward the wild-type level of immune evasion. We did not observe changes with the other *MNT* mutants.

### Genetic disruption of *C. auris MNT5* alters cell wall mannan structure

The cell walls of *C. auris*, like *C. albicans*, contain a higher abundance of N-linked mannans compared to O-linked mannans (23). We hypothesized that highly branched N-mannans may contribute to neutrophil evasion effect in the *C. auris mnt5*∆ mutant. Using transmission electron microscopy, we found a somewhat thicker cell wall for the *mnt5*∆ compared to wild-type stain (Fig. 2B and C). The wild-type strain exhibited a dense, well-defined mannan-rich outer layer. Adjacent to this, we observed a more lucent inner cell wall layer, which has been shown to contain glucan and chitin residues. In contrast, the outer cell walls of the *mnt5*Δ mutant appeared less dense. This loss of the outer layer is consistent with a decrease in cell wall mannan. In addition, these mutant strains had increased electron-lucent layers, suggesting a compensatory increase in glucan and/or chitin.

We next compared the polysaccharides of the *mnt5*∆ and wild-type cell walls using NMR (Fig. 2D). For the *mnt5*∆ cell wall, we observed a general decrease in the mannan content and a relative compensatory increase in the β1,3- and β1,6-glucan components, when compared to wild-type. Comparison of 2D ^1^H-^1^H COSY spectra revealed variations between *C. auris* wild-type and *mnt5*∆ mutant cell walls. For example, two peaks in the α-mannan region (Unk2 and Unk3) were absent in the *mnt5*∆ mutant, while two other peaks (Unk1 and Unk4 peaks) became more intense. Additionally, new signals (Unk6 and Unk7) emerged in the α-mannan region of the *mnt5*∆ mutant, all suggesting structural alterations in the α-mannan of the *mnt5*∆ cell wall with the formation of unique moieties (Fig. 2E; Table S6). The proportion of mannan was lower in mutant (7.5%) compared to the wild-type (20.5%), while the glucan was higher in the *mnt5*∆ mutant (92.4%) compared to the wild-type (79.5%), consistent with a compensatory increase glucan (Fig. 2F).

### The *C. auris mnt5*∆ cell wall displays increased PAMPs

We next questioned how *C. auris* mannosylation pathways might be influencing the interaction with neutrophils. Since the *mnt5*∆ cell wall displayed an overall decrease in mannan content and a structurally altered mannan, we theorized that the overall lack of mannosylation was likely to be contributing to the increased neutrophil engagement observed for these mutants. We reasoned that disruption of the mannoprotein layer could unmask immunostimulatory PAMPs, thus promoting neutrophil engagement. To analyze the cell-surface display of PAMPs in the setting of mannosylation disruption, we utilized immunofluorescence, examining β-glucan and chitin with recombinant dectin-1 and wheat germ agglutinin, respectively (24). The wild-type *C. auris* strain exhibited very little cell surface β-glucan or chitin (Fig. 2F and G). In contrast, the *mnt5Δ* strain displayed both β-glucan and chitin, with quantification of fluorescence revealing over 12- and 6-fold greater PAMP exposure compared to the wild-type strain, respectively.

## DISCUSSION

Cell wall and biofilm matrix mannan play roles in numerous components of *Candida* biology including cell adherence, biofilm formation, and host response (20). Several *Candida* species produce a unique biofilm extracellular matrix mannan-glucan complex that is delivered via EVs (25–28). This complex has been linked to the antifungal resistance biofilm phenotype via sequestration of antifungal (20, 22). While the biofilm matrix mannan component exhibits similarities to cell wall mannan with a α-1,6 backbone and α-1,2 branching (19), the polysaccharide length, branching pattern, and linkage to glucans are unique. In *C. albicans*, several mannan production and modification genes modulate matrix accumulation, and their disruption eliminates the protective antifungal shield, rendering the biofilm communities drug susceptible (20). More recent investigations demonstrated that much of this matrix material is delivered via EVs and that restoration of the matrix via exogenous administration of wild-type EVs returns the resistance biofilm properties (28).

Compared to *C. albicans*, genetic regulation of mannosylation pathways has been largely unexplored for *C. auris*. The present studies identify a role of a putative mannosyltransferase ortholog, *MNT5*, in *C. auris*. Similar to mannan modification enzymes in *C. albicans*, this gene was found to be important for the delivery of a functional biofilm matrix in *C. auris*. Interestingly, the mutant produced similar amounts of mannan but delivered a structurally different polysaccharide. SEM imaging of the *mnt5*∆ mutant exhibited a marked matrix defect. We speculate that this observation implicates these mannan matrix components in the interaction with other critical matrix components such as either the glucan, protein, or eDNA constituents. In *C. albicans*, *MNT5* has been shown to be important for the addition of the α-1,2 mannan branches (16). It is possible that the gene product plays a similar role in *C. auris* and that the α-1,2 mannan structures are critical for deposition and/or incorporation of α-mannans into mannan-glucan complex biofilm extracellular matrix.

Neutrophils are essential for the host response to *Candida* (29, 30). However, neutrophils are less effective at engaging, phagocytosing, and killing *C. auris* when compared to *C. albicans* (21, 24). Cell wall mannan has also been shown to play a role in neutrophil evasion by masking both glucan and chitin PAMPs (21, 24). In the present studies, we find that *MNT5* also participates in cell wall PAMP masking during immune recognition. Like its *C. albicans*, ortholog *C. auris MNT5* also encodes a gene product that decreases cell wall α-mannosylation, perhaps by a decrease in α-1,2 mannan branches, resulting in a compensatory increase in cell wall glucan (16). While it is likely that the susceptibility of *mnt5*∆ to neutrophil phagocytosis is due to the abundance of exposed immunostimulatory surface glucan, it also is possible that fungal recognition may be altered by the structural difference in the cell wall mannans.

While the *MNT5* does not impact fungal growth or fitness in a mouse infection model (not shown), the multifunctional role of this gene in protection from both antifungal drug and host response suggests that the pathway may be an attractive therapeutic target.

## Data Availability

All raw data are available and presented in the supplemental files.

## AFTER EPUB

*[This article was published on 25 February 2025 with an error in the title. The title was corrected in the current version, posted on 10 March 2025.]*

## SUPPLEMENTAL MATERIAL

Supplemental material mbio.00346-25-s0001.pdf Supplemental methods, figures, and tables.

ASM does not own the copyrights to Supplemental Material that may be linked to, or accessed through, an article. The authors have granted ASM a non-exclusive, world-wide license to publish the Supplemental Material files. Please contact the corresponding author directly for reuse.
