# Supplementary material for: Dual function of Candida auris mannosyltransferase, MNT5, in biofilm community protection from antifungal therapy and the host
Source: mBio. 2025 Feb 25;16(4):e00346-25. doi: 10.1128/mbio.00346-25 (PMC11980391; doi:10.1128/mbio.00346-25)
Supplement: Supplemental material — Supplemental methods, figures, and tables. [file mbio.00346-25-s0001.pdf]

Supplemental Materials & Methods

Microorganisms and culture conditions. *Candida auris* B11203, a wild-type Indian isolate from the clade I, was used in this study[1]. A series of individual null deletion mutants targeting the *MNT* mannosyltransferase gene family (mnt1Δ, mnt2Δ, mnt3Δ, mnt4Δ, ktr2Δ, and mnt5Δ) were generated along with their respective gene complements for functional reconstitution (**Table S1**). These mutants were constructed using either nourseothricin or hygromycin B resistance marker-based strategies[2]. Gene replacement cassettes were prepared using PCR-assisted gene splicing by overlap extension (SOE) DNA assembly[3]. At least two independent mutants were created for each targeted gene. Correct integration sites for gene deletions and complementation constructs were verified through routine PCR analysis. The primers used for strain construction and genetic manipulations are detailed in **Table S2**.

Stocks of *C. auris* strains were stored in 15% glycerol frozen at -80°C and routinely maintained on YPD agar plates (1% yeast extract, 2% Bacto™ peptone, 2% dextrose, 2% Bacto™ agar). Liquid cultures were grown in broth YPD (1% yeast extract, 2% Bacto™ peptone, 2% dextrose) rotating at 200 rpm at 30°C. For biofilm growth, strains were cultured in filter-sterilized Roswell Park Memorial Institute medium 1640 (RPMI) containing 0.5% dextrose, and buffered with 4-morpholinepropanesulfonic acid (MOPS), with pH adjusted to 7.0.

Large scale biofilm cultures for cell wall and extracellular matrix isolation. *C. auris* biofilms were grown using a large-scale rolling bottle biofilm model system for 48 hours at 37°C[4]. The culture media were carefully decanted from the polystyrene bottles, and the

resulting fungal biofilms were dislodged from the roller bottle surface with a sterile spatula for further isolation of fungal cell walls and the extracellular matrix. The collected fungal biomass was washed with PBS, centrifuged at 5,000×*g* for 10 min, resuspended in fresh PBS, and subjected to gentle, non-disruptive sonication to remove the extracellular matrix from fungal cells. Sonication was performed using a 6-mm microtip head at 20 kHz with an amplitude of 30% for 8 min, followed by another centrifugation (5,000×*g*, 10 min) to separate the biomass from the matrix. The collected supernatant, containing solubilized extracellular matrix, was filter-sterilized, extensively dialyzed using Slide-A-Lyzer G3 cassettes with a 3,000 molecular weight cutoff, and lyophilized. The collected biofilm fungal cells were resuspended in PBS and transferred to 2-ml microtubes (Sarstedt) containing glass beads. The cells were placed in a bead beater (Mini-Beadbeater, Biospec Products) at full speed for 1-min increments, for a total of 5 min, with 1-min incubations on ice between bead-beating cycles. The contents of the tubes (excluding the glass beads) were transferred to fresh 1.5-ml Eppendorf tubes, rinsed six times with H_2O, and centrifuged at 1,200×*g* to pellet the cell walls[5]. Both the purified extracellular matrix and cell wall preparations were filter-sterilized and stored at -20°C for subsequent experiments.

NMR spectroscopy of *C. auris* biofilm cell walls and extracellular matrices. Approximately 7-12 mg of each cell wall sample was prepared for analysis with ~0.3 mg of DSS as an internal reference. The analysis was done at 343 K on a Bruker Avance III spectrometer (^{1}H, 600 MHz) equipped with a cryoprobe using standard pulse sequences. The chemical shifts are calibrated with DSS (δ_H = 0 ppm and δ_C = 0 ppm). The acquisition parameters are provided in **Table S3**. The spectra were processed and analyzed with

MestReNova v14.2.1-27684 and Bruker Topspin 4.1.3. Recorded ^{1}H and ^{13}C NMR chemical shifts (ppm) of sugar residues tentatively identified in WT fungi cell walls and extracellular matrices are provided in **Table S4** and **Table S5**, respectively. Relative glycan compositions of *C. auris* wild type and *mnt5*Δ mutant cell walls and extracellular matrices are provided in **Table S6** and **Table S7**, respectively.

Biofilm antifungal drug susceptibility assay. The antifungal drug susceptibility of C. auris biofilms was measured using 96-well flat-bottom polystyrene plates[6]. Biofilms were treated with fluconazole, one of the most commonly prescribed antifungal azoles, at a concentration of 1,000 µg/ml. Fungal cell inocula (10^6 cells/ml) were prepared from overnight yeast cultures grown in YPD at 30°C and then diluted in RPMI-MOPS medium containing 0.5% dextrose. Cell counts were determined using an automated Countess™ II cell counter (Invitrogen). One hundred microliters of yeast cell suspension per well were seeded into the plates. After 24 hours, the growth medium was replaced, and fluconazole was added. The plates were then incubated for an additional 24 hours. Biofilms exposed to fluconazole were evaluated using the colorimetric tetrazolium reduction XTT assay. The percent reduction in biofilm growth was calculated by comparing the absorbance of treated biofilms to that of untreated controls. For the XTT assay, XTT (2,3-bis[2-methoxy-4-nitro-5-sulfophenyl]-2H-tetrazolium-5-carboxanilide inner salt) was prepared fresh at a concentration of 0.75 mg/ml. To enhance XTT reduction, 10 µl of 1 mM menadione in acetone and 30 µl of 30% glucose were combined with 960 µl of PBS and added to 9 ml of XTT solution. Absorbance at 492 nm was measured using an automated Cytation 5 imaging reader (BioTek).

The biological impact of exogenous extracellular vesicles on *C. auris* susceptibility to fluconazole was evaluated using 96-well plates. Fungal cell inocula (10^6 cells/ml) were prepared from overnight yeast cultures grown in YPD medium at 30°C and diluted in RPMI-MOPS medium based on cell counts obtained with an automated Countess™ II cell counter (Invitrogen). Each well was seeded with 100 µl of yeast cell suspension, followed by the addition of extracellular vesicles and incubation for 24 hours at 37°C. After incubation, the growth media were replaced, fresh extracellular vesicles were re-applied, and cells were treated with fluconazole (1,000 µg/mL). Biofilm growth in cultures with and without exogenous extracellular vesicles was assessed using the XTT assay as described above.

Scanning electron microscopy of *C. auris* biofilms. The surface of *Candida* biofilms grown in 6-well plates was imaged using scanning electron microscopy (SEM)[2]. Briefly, 40 µl of an inoculum containing 10^8 cells/ml in RPMI containing 0.5% dextrose was added to coverslips and incubated at 37°C for 60 minutes. One ml of RPMI was then added to each well, and the plates were incubated at 37°C for 20 hours. Following incubation, 1 ml of fixative (4% formaldehyde, 1% glutaraldehyde in PBS) was added to each well, and the plates were incubated at 4°C overnight. The coverslips were then washed with PBS and incubated in 1% OsO_4 for 30 minutes. Samples were serially dehydrated in ethanol (30% to 100%) before undergoing critical point drying to ensure complete dehydration. After dehydration, the samples were coated with palladium-gold and imaged using a LEO 1530 SEM. Image compilation was performed using Adobe Photoshop 2022 (v. 23.2.2).

Human neutrophil collection. Human blood was obtained from volunteer donors who provided informed written consent, in accordance with a protocol approved by the Institutional Review Board of the University of Wisconsin-Madison. Neutrophils were isolated as previously described using the MACSxpress negative antibody selection kit and purified with the MACSxpress erythrocyte depletion kit (Miltenyi Biotec, Inc., Auburn, CA). Isolated neutrophils were resuspended in RPMI 1640 (phenol red-free), supplemented with glutamine (0.3 mg/ml) and 2% heat-inactivated fetal bovine serum (FBS). Neutrophil incubations were conducted at 37°C with 5% CO_2[7].

Phagocytosis assays. *Candida* strains were stained with calcofluor white (100 µg/ml) in the dark for 10 minutes at room temperature, then washed three times in DPBS. A suspension of 4×10^6 stained cells was added to a tissue-cultured microslide (Ibidi). Staining with calcofluor white had no effect on the viability of *C. auris* wild type (WT) or *mnt5*Δ strains, with stained yeast growing to 101.8 ± 9.5%, 110.8 ± 24.8%, and 95.5 ± 13.5% of the growth observed in unstained controls, respectively. Neutrophils were fluorescently labeled with calcein acetoxymethyl (AM) at 0.5 µg/ml (Thermo Fisher Scientific, Waltham, MA) in the dark for 10 minutes at room temperature. One million neutrophils were then added to the microslide wells. After 1 hour of incubation, images were captured using a Nikon Eclipse-TI2 inverted microscope equipped with an ORCA-Flash 4.0 LT sCMOS camera, a TI2-S-SS-E motorized stage, a stage-top TIZW series Neco incubation system (Tokai Hit), and NIS Elements imaging software. DAPI (4',6-diamidino-2-phenylindole) (Ex 378/52: Em 447/60 nm) and fluorescein isothiocyanate (FITC) (Ex 466/40: Em 525/50) filters were used for imaging. Subsequently, neutrophils involved in phagocytosis of *C. auris* yeast were enumerated, and the percentage of

engaged neutrophils was calculated using the following formula "(engaged neutrophils in frame/total neutrophils in frame) × 100" as previously described.

Fluorescent labeling of *C. auris* cell wall components. *Candida* cells were adjusted to 1 × 10^7 cells/ml and suspended in 1 ml of a 4°C blocking solution, which contained 0.5% bovine serum albumin (BSA), 5% heat-inactivated fetal bovine serum (HI-FBS), 5 mM EDTA, and 2 mM NaN_3 in 1× DPBS. The suspension was incubated for 30 minutes at room temperature on a MACSmix tube rotator (Miltenyi Biotec, Inc., Auburn, CA) at 9 rpm. Cells were then collected by centrifugation, washed twice with cold flow cytometry washing solution (0.5% BSA, 5 mM EDTA, and 2 mM NaN_3 in DPBS), and labeled with Fc:dectin-1 protein (Fc [human]:dectin-1 [mouse], recombinant, Adipogen) at 1 µg/ml in blocking solution for 1 hour on ice. Blocking buffer-only controls were included for each strain. The cells were washed three times with washing solution, then resuspended in a 1:200 dilution of Alexa Fluor 488-conjugated antihuman IgG Fc antibody (Fc1 488; Biolegend) in blocking solution. The cells were incubated on ice in the dark for 45 minutes, washed three times with washing solution, and resuspended to 1 × 10^7 cells/ml in washing solution. The cell suspension (150 µl) was added in triplicate to wells of a black 96-well microtiter plate (Costar), and fluorescence intensity was quantified using a microplate reader at 488/519 nm[5].

To analyze chitin exposure, wheat germ agglutinin conjugated to fluorescein isothiocyanate (WGA-FITC) was used to label exposed chitin. *Candida* cells were adjusted to 1 × 10^7 cells/ml and suspended in 1 ml of 4°C DPBS containing 2% BSA (wt/vol). The suspension was incubated for 30 minutes at room temperature on a MACSmix tube rotator (Miltenyi Biotec, Inc., Auburn, CA) at 9 rpm. Cells were then

collected by centrifugation, washed twice with cold DPBS containing 0.05% Tween 20, and labeled with 0.1 mg/ml WGA-FITC in tubes containing DPBS with 1% BSA. Unstained controls were included for each strain. Cells were incubated in the dark at room temperature for 1 hour, washed three times with DPBS containing 0.05% Tween 20, and resuspended to 1 × 10^7 cells/ml in DPBS with 0.05% Tween 20. The cell suspension (150 µl) was added in triplicate to wells of a black 96-well microtiter plate (Costar), and fluorescence intensity was quantified in a microplate reader at 488/519 nm[5].

Fluorescent microscopy was used to visualize β-glucan-labeled and chitin-labeled yeast. Briefly, the fluorescently labeled yeast were added to wells of a microslide (Ibidi) and imaged using a Nikon Eclipse-TI2 inverted microscope equipped with an ORCA-Flash 4.0 LT sCMOS camera, a TI2-S-SS-E motorized stage, a stage-top TIZW series Neco incubation system (Tokai Hit), and NIS Elements imaging software. Imaging was performed using the FITC filter at 30× magnification[5].

Transmission electron microscopy. Transmission electron microscopy (TEM) was used to examine the cell walls of *C. auris* strains, as previously described[8]. Briefly, cells were fixed in 4% formaldehyde and 2% glutaraldehyde, followed by post-fixation with 1% osmium tetroxide and 1% potassium ferricyanide. The cells were then stained with 1% uranyl acetate, dehydrated through a graded series of ethanol solutions, and embedded in Spurr’s resin. Thin sections (70 nm) were cut and placed on copper grids, poststained with 8% uranyl acetate in 50% methanol and Reynolds' lead citrate. The samples were analyzed using a Philips CM 120 transmission electron microscope. Cell wall lengths were manually measured using Fiji.

Statistics. Data sets of equal or different sample sizes were analyzed using the nonparametric Kruskal-Wallis one-way analysis of variance, followed by uncorrected Dunn’s multiple comparisons, without prior elimination of outliers. Data were processed using GraphPad Prism 10 for Windows 64-bit (version 10.4.0 (621)).

Table S1. *Candida auris* strain genotypes used in this study

Strain /Gene systematic name	Phenotype	Genetic makeup	Reference
B11203	Reference strain	*Candida auris* wild type Indian isolate of the clade I	[1]
RZU059 C3_01810C_A/MNT1	*mnt1* null deletion Nat1^{+}	*mnt1*Δ::NAT1	This work
RZU081	*mnt1* complement HygB^{+}	*mnt1*Δ::NAT1 / NAT1::MNT1-HygB	This work
RZU063 C3_01830C_A/MNT2	*mnt2* null deletion Nat1^{+}	*mnt2*Δ::NAT1	This work
RZU089	*mnt2* complement HygB^{+}	*mnt2*Δ::NAT1 / NAT1::MNT2-HygB	This work
RZU069 CR_05290W_A/MNT3	*mnt3* null deletion Nat1^{+}	*mnt3*Δ::NAT1	This work
RZU097	*mnt3* complement HygB^{+}	*mnt3*Δ::NAT1 / NAT1::MNT3-HygB	This work
RZU071 CR_04800W_A/MNT4	*mnt4* null deletion Nat1^{+}	*mnt4*Δ::NAT1	This work
RZU105	*mnt4* complement HygB^{+}	*mnt4*Δ::NAT1 / NAT1::MNT4-HygB	This work
RZU075 C2_04560W_A/KTR2/MNT4b	*mnt4b* null deletion Nat1^{+}	*mnt4b*Δ::NAT1	This work
RZU113	*mnt4b* complement HygB^{+}	*mnt4b*Δ::NAT1 / NAT1::MNT4b-HygB	This work
RZU079 B9J08_003123/MNT5	*mnt5* null deletion Nat1^{+}	*mnt5*Δ::NAT1	This work
RZU121	*mnt5* complement HygB^{+}	*mnt5*Δ::NAT1 / NAT1::MNT5-HygB	This work

Table S2. PCR primers used in this study

Target Gene Primer Name	Function	Sequence
CR_MNT1 5' FLANK F	deletion	CTTCTCCAAGATCAAACGCC
CR_MNT1 5' FLANK R	deletion	cacggcgcgcctagcagcgg CCTTTTCCTTGTCGATCCAC
CR_MNT1 3' FLANK F	deletion	gtcagcggccgcatccctgc CTTCTGTGACATTGACTACGA
CR_MNT1 3' FLANK R	deletion	TTTCCCATCCCTTAGGTCTC
CR_MNT1 NESTED F	deletion	TTGTGTTGATCTTCTGTGGC
CR_MNT1 NESTED R	deletion	ATCCGTGGAGAGTGAAGTAC
CR_MNT1 INTERNAL F	deletion	ACGCATCCAACCCTAATCTA
CR_MNT1 INTERNAL R	deletion	AGTTATGGAAAGGAACGTGC
CR_MNT1 5' FLANK F	deletion	TTTTGTCTTCCTCCTCTCCT
CR_MNT1 5' FLANK R	deletion	GTGCGGCCATCAAAATGTAT
CR_MNT1 3' FLANK F	deletion	AAGTTAAGTGCGCAGAAAGT
CR_MNT1 3' FLANK R	deletion	GAATTCTGAAAATGGGGCCT
CR_MNT1 compl 5' FLANK F/ORF	complementation	TTCACCTTCAGAGGGTACAGT
CR_MNT1 compl 5' FLANK R/ORF	complementation	cacggcgcgcctagcagcgg CCACGAAAGACACCCTAATTT
CR_MNT1 compl 3' FLANK F	complementation	gtcagcggccgcatccctgc CTAGCAAAACTTCGATAACTGC
CR_MNT1 compl 3' FLANK R	complementation	AGGGTGGTCTATGACGAGTT
CR_MNT1 compl NESTED F	complementation	GGGAAGAAATACTACGAGGCTA
CR_MNT1 compl NESTED R	complementation	AGGACAAGTCAAGAAAGTGCTA
CR_MNT1 compl UPSTREAM CHECK F	complementation	GAGTTCAGATATGAGCACAGGT
CR_MNT1 compl UPSTREAM CHECK R	complementation	GTAGTTAGAGGATGGGGAAGAG
CR_MNT1 compl DOWNSTREAM CHECK F	complementation	GGATAGAGCCAACGATAAGAAC
CR_MNT1 compl DOWNSTREAM CHECK R	complementation	CTTCACTCAAATTGTGGGTATC
CR_MNT2 5' FLANK F	deletion	TGCTATACTTGATCTCGGCC
CR_MNT2 5' FLANK R	deletion	cacggcgcgcctagcagcgg CTCCTTAGGAATCTCGCCAT
CR_MNT2 3' FLANK F	deletion	gtcagcggccgcatccctgc TGTGTCGATTTGAAAGTGGA
CR_MNT2 3' FLANK R	deletion	CCCGACATCATGGAAATAGT
CR_MNT2 NESTED F	deletion	GACCGTTGTTTCAGACTTCA
CR_MNT2 NESTED R	deletion	ATCGCCCCATCTCTCGTA
CR_MNT2 INTERNAL F	deletion	CATCAAGGACTCGTCTCAGG
CR_MNT2 INTERNAL R	deletion	CGGTCTGGTCAAGGTATTCA
CR_MNT2 5' FLANK F	deletion	TCCGTTCCTGAGGCTTATTA
CR_MNT2 5' FLANK R	deletion	AATTCAACGCGTCTGTGAG
CR_MNT2 3' FLANK F	deletion	TGCCCAGATGCGAAGTTAA

CR_MNT2 3' FLANK R	deletion	TTGGTCCTAGTGCTTTCGC
CR_MNT2 compl 5' FLANK F/ORF	complementation	CTCATCTTCCCAGGAATTGG
CR_MNT2 compl 5' FLANK R/ORF	complementation	cacggcgcgcctagcagcgg TCTACACGCACAATCACCAGA
CR_MNT2 compl 3' FLANK F	complementation	gtcagcggccgcatccctgc AGCGCGTGTAGTGAAAGACA
CR_MNT2 compl 3' FLANK R	complementation	AGATGGCGAATCTGACCAAC
CR_MNT2 compl NESTED F	complementation	TGCAACCAATGTGAAGTGCT
CR_MNT2 compl NESTED R	complementation	GCGTTTGATCTTGGAGAAGC
CR_MNT2 compl UPSTREAM CHECK F	complementation	CGCAATAACACAATGCTGCT
CR_MNT2 compl UPSTREAM CHECK R	complementation	TCGTGTCGAGTTGTTCTTGG
CR_MNT2 compl DOWNSTREAM CHECK F	complementation	CGTCACGGGTATTTTCTCTTG
CR_MNT2 compl DOWNSTREAM CHECK R	complementation	GTTCTTGGGAATTGCCTGTG
CR_MNT3 5' FLANK F	deletion	GCGGTTGTGCTCTTCTTTAA
CR_MNT3 5' FLANK R	deletion	cacggcgcgcctagcagcgg ACTTCCCCAATGTTCCTTTG
CR_MNT3 3' FLANK F	deletion	gtcagcggccgcatccctgc TATTGGCTACGATGTGTTCC
CR_MNT3 3' FLANK R	deletion	TACAAGGGTACACAGCTGAG
CR_MNT3 NESTED F	deletion	CAATAGATGGTTAGTGGACGA
CR_MNT3 NESTED R	deletion	AGTAGCCTATATCGCCGAAC
CR_MNT3 INTERNAL F	deletion	TCGAAAGCTACCTTGAACAG
CR_MNT3 INTERNAL R	deletion	CCAGAGACGGCTTTTGTTAA
CR_MNT3 5' FLANK F	deletion	CTGATGAAAGTAGACGACAGA
CR_MNT3 5' FLANK R	deletion	TGGGGATGTATGGGCTAAAT
CR_MNT3 3' FLANK F	deletion	GCCCAGATGCGAAGTTAAG
CR_MNT3 3' FLANK R	deletion	ATTGGCGTGAAATCTCTGAA
CR_MNT3 compl 5' FLANK F/ORF	complementation	AGCGGGAAGTGGCTGTTC
CR_MNT3 compl 5' FLANK R/ORF	complementation	cacggcgcgcctagcagcgg AAAATAATACCTTACCCTCTTTGTTGA
CR_MNT3 compl 3' FLANK F	complementation	gtcagcggccgcatccctgc TTTTAATCAGAGGGTTTGACG
CR_MNT3 compl 3' FLANK R	complementation	ATGCAGAGTAATATGAGCGAAT
CR_MNT3 compl NESTED F	complementation	GTACTAGGCAAGGAAGAAGCA
CR_MNT3 compl NESTED R	complementation	GAGTTTTTCCTTGTAGGATGGT
CR_MNT3 compl UPSTREAM CHECK F	complementation	GCCTCTAAATAACATCAACGAG
CR_MNT3 compl UPSTREAM CHECK R	complementation	CGTATATACAGAAACGCAGGTT
CR_MNT3 compl DOWNSTREAM CHECK F	complementation	TTTATTATTTTCGTCACGGGTA
CR_MNT3 compl DOWNSTREAM CHECK R	complementation	GCTTAGCAGTTCTAATGGTGTT
CR_MNT4 5' FLANK F	deletion	GATGCTTCACCTCCACTAGA
CR_MNT4 5' FLANK R	deletion	cacggcgcgcctagcagcgg ACTCAGGCACCTTCATATCA

CR_MNT4 3' FLANK F	deletion	gtcagcggccgcatccctgc AACCACGGTACCAAGTTCTA
CR_MNT4 3' FLANK R	deletion	ACACCAGTAGTCATCCAGAG
CR_MNT4 NESTED F	deletion	AGCAACAGCATACAAGTGAA
CR_MNT4 NESTED R	deletion	GCAACTCCACCAATTGATCT
CR_MNT4 INTERNAL F	deletion	AAAGCTTATCAAGACAGGCC
CR_MNT4 INTERNAL R	deletion	GGGACGTACTCAATATTGCC
CR_MNT4 5' FLANK F	deletion	CTCTGTTTACCTTCTACGCG
CR_MNT4 5' FLANK R	deletion	ATGGGGATGTATGGGCTAAA
CR_MNT4 3' FLANK F	deletion	AAGTTAAGTGCGCAGAAAGT
CR_MNT4 3' FLANK R	deletion	ATGCCTTGTTTTGGTTTCAC
CR_MNT4 compl 5' FLANK F/ORF	complementation	GTAACTTCTCTTCCTCCTCCAT
CR_MNT4 compl 5' FLANK R/ORF	complementation	cacggcgcgcctagcagcgg AAATGTATACACTGGCATGCTT
CR_MNT4 compl 3' FLANK F	complementation	gtcagcggccgcatccctgc GTCGTGCACATAGAGAACAAG
CR_MNT4 compl 3' FLANK R	complementation	CCTTAAATGTTTCGTATCGTCT
CR_MNT4 compl NESTED F	complementation	ACAATCTGGCTCCACATACTAA
CR_MNT4 compl NESTED R	complementation	AACAAGGCTCAATTGATATAGAA
CR_MNT4 compl UPSTREAM CHECK F	complementation	AATAACGCAAAAGATAAGACGA
CR_MNT4 compl UPSTREAM CHECK R	complementation	TGGGGCAGTTATAGTGAGATAC
CR_MNT4 compl DOWNSTREAM CHECK F	complementation	ACCCAAGGCATTTCTATATCTT
CR_MNT4 compl DOWNSTREAM CHECK R	complementation	TTTTCCACATCTCTCTTCAATC
CR_MNT4B 5' FLANK F	deletion	CCTTTGGTTTTGTTGATCGC
CR_MNT4B 5' FLANK R	deletion	cacggcgcgcctagcagcgg AGCTTCATCCACAAAGTCTG
CR_MNT4B 3' FLANK F	deletion	gtcagcggccgcatccctgc TAAATACTACTGGCGCTTGG
CR_MNT4B 3' FLANK R	deletion	GTAATCGCTATTGTGGTCCG
CR_MNT4B NESTED F	deletion	AGTCTTATCGTGGTGCATTC
CR_MNT4B NESTED R	deletion	GCGTGTGATGATAGCCAATA
CR_MNT4B INTERNAL F	deletion	GCTAAGCACTTTTCCCTGAA
CR_MNT4B INTERNAL R	deletion	ACTTTCCGGTAGAGTCCAAA
CR_MNT4B 5' FLANK F	deletion	GAAAATCCCTCCACAAACCA
CR_MNT4B 5' FLANK R	deletion	GTGCGGCCATCAAAATGTAT
CR_MNT4B 3' FLANK F	deletion	AAGTTAAGTGCGCAGAAAGT
CR_MNT4B 3' FLANK R	deletion	ATACCGGGATCAATAGGCTT
CR_MNT4B compl 5' FLANK F/ORF	complementation	ATGAGAATTCCTCCAAAGAATG
CR_MNT4B compl 5' FLANK R/ORF	complementation	cacggcgcgcctagcagcgg TATGATGGGTTGTTTCGATATG
CR_MNT4B compl 3' FLANK F	complementation	gtcagcggccgcatccctgc AGCTCGCTTATCTCGGTTAT

CR_MNT4B compl 3' FLANK R	complementation	TCTTACCATCCTTAGTGTCTGG
CR_MNT4B compl NESTED F	complementation	CGTCTCTCGTTTCTCCATC
CR_MNT4B compl NESTED R	complementation	CTTCTGTGGAGACAAATCTTGA
CR_MNT4B compl UPSTREAM CHECK F	complementation	GTACAAGGGAAATGCTTACAAT
CR_MNT4B compl UPSTREAM CHECK R	complementation	GAAATCAGTGGGCTTTGTTC
CR_MNT4B compl DOWNSTREAM CHECK F	complementation	GGGTATTTTCTCTTGTTTGATG
CR_MNT4B compl DOWNSTREAM CHECK R	complementation	GAACTCCTTGGCGTAGTCTT
CR_MNT5 5' FLANK F	deletion	ACTGATCCCTACACAAAGCT
CR_MNT5 5' FLANK R	deletion	cacggcgcgcctagcagcgg TTCTGTATGAGCGAGAAGGA
CR_MNT5 3' FLANK F	deletion	gtcagcggccgcatccctgc TTCAGTACGATCCATTCACC
CR_MNT5 3' FLANK R	deletion	TCTGGGTAGACAACTTGGC
CR_MNT5 NESTED F	deletion	GCATCCTAAAACCAGCTCTG
CR_MNT5 NESTED R	deletion	GCCATCAAATGCCTTATCTCT
CR_MNT5 INTERNAL F	deletion	GTATAATCCCGGGCACAAAA
CR_MNT5 INTERNAL R	deletion	AAGAAGCAAAGCAAGTCCAA
CR_MNT5 5' FLANK F	deletion	TGCAATAAACCGAATCCCAG
CR_MNT5 5' FLANK R	deletion	AATTCAACGCGTCTGTGAG
CR_MNT5 3' FLANK F	deletion	TCGCCTCGACATCATCTG
CR_MNT5 3' FLANK R	deletion	TTATGAGATAAAGGAGCGCC
CR_MNT5 compl 5' FLANK F/ORF	complementation	ACTGTGTAATCGAAATTCTTGC
CR_MNT5 compl 5' FLANK R/ORF	complementation	cacggcgcgcctagcagcgg TAACTTGGATAATTTTGGATGC
CR_MNT5 compl 3' FLANK F	complementation	gtcagcggccgcatccctgc TAGATACAAGAAGATGGGGAAA
CR_MNT5 compl 3' FLANK R	complementation	AGACTTAACCATCTCAGCAAAG
CR_MNT5 compl NESTED F	complementation	AAAACTTGAGGATGTCACAGAG
CR_MNT5 compl NESTED R	complementation	TGGAACTTTCAACTTTAGGAGA
CR_MNT5 compl UPSTREAM CHECK F	complementation	CCGTAGCCAACTGTCTGA
CR_MNT5 compl UPSTREAM CHECK R	complementation	ATTACTTCAACAGTGAAGGAGGT
CR_MNT5 compl DOWNSTREAM CHECK F	complementation	TTTATTATTTTCGTCACGGGTA
CR_MNT5 compl DOWNSTREAM CHECK R	complementation	CGAACTTTTTCAACTTTCTTTG

Table S3. NMR spectroscopy acquisition parameters

Sample type	Experiment	Parameters at 343 K in 600 MHz spectrometer								
		d1 (s)	NS	td2	td1	aq2 (s)	aq1 (s)	sw2 (ppm)	sw1 (ppm)	Time
Cell walls	1D ^{1}H	30	8	65536	-	2.03	-	20	-	6 min
	2D COSY	1.5	6	2048	256	0.09	0.05	18	8	42 min
	2D HSQC	1.5	20	2048	256	0.09	0.006	18	124	2.20hr
	2D TOCSY	1.5	8	2048	256	0.09	0.02	18	9	1hr
Extracellular matrices	1D ^{1}H	60	8	50000	-	2.6	-	16	-	12 min
	2D COSY	1.5	2	2048	256	0.09	0.05	18	8	14 min
	2D HSQC	1.5	16	2048	256	0.09	0.007	18	120	2.20 h

Table S4. Recorded ^{1}H and ^{13}C NMR chemical shifts (ppm) of sugar residues identified in *C. auris* cell walls

Residue			1	2	3	4	5	6	6`	Reference
β-Man-(1→2)-α-Man-(1→	A	^{1}H	5.16	4.318	-	-	-	-	-	[5,9,10]
		^{13}C	98.6	-	-	-	-	-	-	
β-Man-(1→2)-α-Man-(1→2)	B	^{1}H	5.16	4.254	-	-	-	-	-	
		^{13}C	98.78	-	-	-	-	-	-	
t-α-Man-(1→3	C	^{1}H	5.15	4.16	-	-	-	-	-	
		^{13}C	99.3	-	-	-	-	-	-	
t-α-Man-(1→3	D	^{1}H	5.15	4.119	-	-	-	-	-	
		^{13}C	99.4	-	-	-	-	-	-	
2,6)-α-Man-(1→2)-Man	E	^{1}H	5.169	4.065	-	-	-	-	-	
		^{13}C	98.71	-	-	-	-	-	-	
2,6)-α-Man-(1→2)-Man	F	^{1}H	5.13	4.04	-	-	-	-	-	
		^{13}C	99.1	-	-	-	-	-	-	
2,6)-α-Man-(1→2)-Man	G	^{1}H	5.16	4.02	-	-	-	-	-	
		^{13}C	98.7	-	-	-	-	-	-	
t-β-Man-(1→2)-Man	H	^{1}H	4.95	4.11	-	-	-	-	-	
		^{13}C	-	-	-	-	-	-	-	
t-β-Man-(1→2)-Man	I	^{1}H	4.94	4.05	-	-	-	-	-	
		^{13}C	-	-	-	-	-	-	-	
?)-α-Man-(1→	J	^{1}H	5.08	3.86	-	-	-	-	-	
		^{13}C	100.6	-	-	-	-	-	-	
?)-α-Man-(1→	K	^{1}H	5.08	3.81	-	-	-	-	-	
		^{13}C	99.71	-	-	-	-	-	-	
?)-α-Man-(1→	L	^{1}H	5.10	3.91	-	-	-	-	-	
		^{13}C	100.5	-	-	-	-	-	-	
Reducing end/free α-Hex*p*	M	^{1}H	4.91	3.29	-	-	-	--		[11]
		^{13}C	92.6	-	-	-	-	-	-	
Reducing end/free α-Hex*p*	N	^{1}H	4.93	3.16	-	-	-	-	-	
		^{13}C	91.7	-	-	-	-	-	-	
Reducing end	O	^{1}H	4.91	3.58	-	-	-	-	-	
/free α-Hex*p*		^{13}C	93.67	-	-	-	-	-	-	
Reducing end	P	^{1}H	4.947	3.49	-	-	-	-	-	
/free α-Hex*p*		^{13}C	93.67	-	-	-	-	-	-	
(3,6)-β-Glc*p*-(1→	Q	^{1}H	4.54	3.27	-	-	-	-	-	[12]
		^{13}C	102.4	76.5	-	-	-	-	-	
3)-β-Glc*p*-(1→3)	R	^{1}H	4.54	3.31	3.48	-	-	3.69	-	
		^{13}C	102.4	-	-	-	-	-	-	
6)-β-Glc*p*-(1→6)	S	^{1}H	4.286	3.04	3.63	3.74	3.87	-	-	
		^{13}C	102.7	-	-	-	-	-	-	
β-Glc*p*-reducing	T	^{1}H	4.305	2.96	-	-	-	-	-	[11]
end/ free β-Glc*p*		^{13}C	96.4	-	-	-	-	-	-	
Glycogen	U	^{1}H	5.13	3.34	-	-	-	-	-	[13-17]

4)-α-Glc*p*-(1→4)		^{13}C	99.0	-	-	-	-	-	-
4,6)-α-Glc*p*-(1→4)	V	^{1}H	5.09	3.33	-	-	-	-	-
		^{13}C	99.71	-	-	-	-	-	-
6)-α-Glc*p*-(1→6)	W	^{1}H	5.052	3.285	-	-	-	-	-
		^{13}C	99.9	-	-	-	-	-	-
Unidentified peaks									
Unk1		^{1}H	4.62	4.26					
		^{13}C		-	-	-	-	-	-
Unk2		^{1}H	4.66	3.98					
		^{13}C	98.3	-	-	-	-	-	-
Unk3		^{1}H	4.75	3.81					
		^{13}C	99.9	-	-	-	-	-	-
Unk4		^{1}H	4.73	4.21					
		^{13}C	100.1	-	-	-	-	-	-
Unk5		^{1}H	4.98	3.767					
		^{13}C	99.5	-	-	-	-	-	-
Unk6		^{1}H	5.12	4.39					
		^{13}C	-	-	-	-	-	-	-
Unk7		^{1}H	5.20	4.41					
		^{13}C	-	-	-	-	-	-	-

Table S5. Recorded ^{1}H and ^{13}C NMR chemical shifts (ppm) of sugar residues identified in C. auris extracellular matrices

Residue			1	2	Reference
[β-Man-(1→2)]n-α-Man-(1→ PO_4	A	^{1}H	5.55	4.18	[10,12]
		^{13}C	96.8		
α-Man-(1→PO_4	B	^{1}H	5.45	4.01	
		^{13}C	98.9		
α-Man-(1→2)-α-Man-(1→3)-α-Man-(1→	C	^{1}H	5.35	4.13	
		^{13}C	102.4		
α-Man-(1→2)-α-Man-(1→3)-α-Man-(1→	D	^{1}H	5.34	4.06	
		^{13}C			
α-Man-(1→2)-α-Man-(1→2)	E	^{1}H	5.25	4.11	
		^{13}C	103.2		
α-Man-(1→2)-α-Man-(1→2)	F	^{1}H	5.27	4.097	
		^{13}C	102.9		
β-Man-(1→2)-[β-Man-(1→2)]n-α-Man-(1→2)	G	^{1}H	5.16	4.25	
		^{13}C	102.6		
→6)[α-Man-(1→2)-α-Man-(1→2)]-α-Man-(1→	H	^{1}H	5.06	4.02	
		^{13}C	101.0		
α-Man-(1→2) *or*	I	^{1}H	5.05	4.084	
→6)[α-Man-(1→2)-α-Man-(1→2)]-α-Man-(1→6)		^{13}C	101.2		
α-Man-(1→3)-α-Man-(1→2)-α-Man-(1→	J	^{1}H	5.04	4.19	
		^{13}C	104.7		
α-Man-(1→6)	K	^{1}H	4.92	4.00	
		^{13}C	102.4		
α-Man-(1→6)	L	^{1}H	4.908	3.98	
		^{13}C	102.3		
β-Man-(1→2)-[β-Man-(1→2)]n-β-Man-(1→2)-α-Man-	M	^{1}H	4.89	4.28	
(1→PO_4		^{13}C	103.7		
β-Man-(1→2)-β-Man-(1→2)-α-Man-(1→2	N	^{1}H	4.84	4.17	
or same with terminal (1→PO_4		^{13}C	103.6		
β-Man-(1→2)-[β-Man-(1→2)]n-β-Man-(1→2)-α-Man-	O	^{1}H	4.84	4.24	
(1→2		^{13}C	101.7		
β-Man-(1→2)-α-Man-(1→PO_4	P	^{1}H	4.84	4.05	
		^{13}C	103.6		
3)-β-Glc*p*-(1→3)	Q	^{1}H	4.72	3.38	[18,19]
		^{13}C	105.5		
3,6)-β-Glc*p*-(1→3)	R	^{1}H	4.72	3.33	
		^{13}C	105.5		
unknown, possibly not carbohydrate	S	^{1}H	4.619	3.785	
		^{13}C	-		
3,6)-β-Glc*p*-(1→6)	T	^{1}H	4.551	3.535	
		^{13}C	105.3		
6)-β-Glc*p*-(1→6)	U	^{1}H	4.514	3.347	
		^{13}C	105.6		
Glycogen	V	^{1}H	5.355	3.638	[12,15-17]
4)-α-Glc*p*-(1→4)		^{13}C	102.5		
4,6)-α-Glc*p*-(1→4)	W	^{1}H	5.356	3.599	
		^{13}C	102.5		
	X	^{1}H	5.395	3.659	
		^{13}C	102.9		
6)-α-Glc*p*-(1→6)	Y	^{1}H	4.97	3.59	
		^{13}C	101.5		
New peaks from MNT5Δ cell walls					
β-Man-(1→2)-[β-Man-(1→2)]n-α-Man-(1→3		^{1}H	5.24	4.22	[10,12]
β-Man-(1→2)-[β-Man-(1→2)]n		^{1}H	4.91	4.15	
→2-β-Man-(1→2		^{1}H	4.90	4.39	

Table S6. Relative abundance of *C. auris* cell walls glycan residues identified in the 1D ^{1}H NMR and 2D COSY spectra based on integration of the 1D anomeric peaks and the 2D H1-H2 cross peaks.

Integration region			Relative abundance [%]					
			WT			MNT5Δ		
1D ^{1}H NMR	Residue	Peak labels	Overall	Man+β-Glc	β-Glc	Overall	Man+β-Glc	β-Glc
5.19-5.12	-6/2/3)-α-Man-(1→ +glycogen	A-G+U	7.8	20.5	-	4.0	7.9	-
4.56-4.51	-3)-β-Glc-(1→3)	R, Q	13.8	34.2	43.1	22.2	44.2	48.0
4.30-4.27	6)-β-Glc-(1→6	T	17.2	45.2	56.9	23.9	47.8	51.9
		Overall total		100	100		100	100
4.32-4.30	β-Glc reducing/ free	S	18.1			13.1		
4.94-4.92	Reducing end/free α-Hex*p*	N, P	3.5			2.2		
4.92-4.90	Reducing end/free α-Hex*p*	O, M	11.1			9.1		
4.84-4.60	Unknown structures	Unk	29.0			25.5		
		Overall total	100			100		

Table S7. Relative abundance of glycan residues in *C. auris* extracellular matrix identified in the 1D ^{1}H NMR spectra based on integration of the anomeric peaks. Ratios between mannan residues only and β-glucan residues only are in green and blue shaded cells, respectively.

1D ^{1}H NMR (d1=30s)				Relative abundance [%]			
				WT		MNT5	
Integration region	Type	Peak labels	Residue	Overall	By type	Overall	By type
5.57-5.52	Mannan	A	[β-Man-(1→2)]n-α-Man-(1→ PO_4	1.4	3.6	1.6	2.3
5.48- 5.43		B	α-Man-(1→PO_4	0.4	1.0	0.8	1.1
5.29- 5.20		E, F	α-Man-(1→2)-α-Man-(1→2)	5.4	14.3	14.1	19.8
5.19 .. 5.13		G	β-Man-(1→2)-[β-Man-(1→2)]n-α-Man-(1→2)	5.1	13.3	8.8	12.4
5.10- 5.00		H, I, J	6)[α-Man-(1→2)-α-Man-(1→2)]-α-Man-(1→ /<br>Man-(1→2) *or*<br>→6)[α-Man-(1→2)-α-Man (1→2)]-α-Man-(1→6) /<br>α-Man-(1→3)-α-Man-(1→2)-α-Man-(1→	9.9	26.3	18.8	26.5
4.94- 4.88		K, L, M	α-Man-(1→6) /<br>β-Man-(1→2)-[β-Man-(1→2)]n-β-Man-(1→2)-α-Man-(1→PO_4	5.8	15.3	8.9	12.6
4.86- 4.81		N, O, P	β-Man-(1→2)-β-Man-(1→2)-α-Man-(1→2 or terminal (1→PO_4<br>β-Man-(1→2)-[β-Man-(1→2)]n-β-Man-(1→2)-α-Man-(1→2<br>β-Man-(1→2)-α-Man-(1→PO_4	9.9	26.2	17.9	25.3
			Mannan only		100		100
4.75- 4.68	β -glucan	Q, R	3(,6))-β-Glc*p*-(1→3)	0.6	19.3	0.3	19.1
4.54- 4.49		T, U	6)- β -Glc*p*-(1→6)	2.4	80.7	1.3	80.7
			β -glucan only		100		100

Fig S1 The percent reduction in biofilm formation was measured following treatment with fluconazole (1000 μg/ml), compared to untreated biofilms in wild-type and *mnt1*Δ*, mnt2*Δ*, mnt3*Δ*, mnt4*Δ*, mnt4b*Δ*,* and *mnt5*Δ. Each dot represents an independent biological replicate and reflects the mean of 8 technical replicates. Error bars denote standard deviation. A non-parametric Kruskal–Wallis one-way analysis of variance with an uncorrected Dunn's multiple comparison test was performed, with a significant *p*-value indicated as <0.0001.

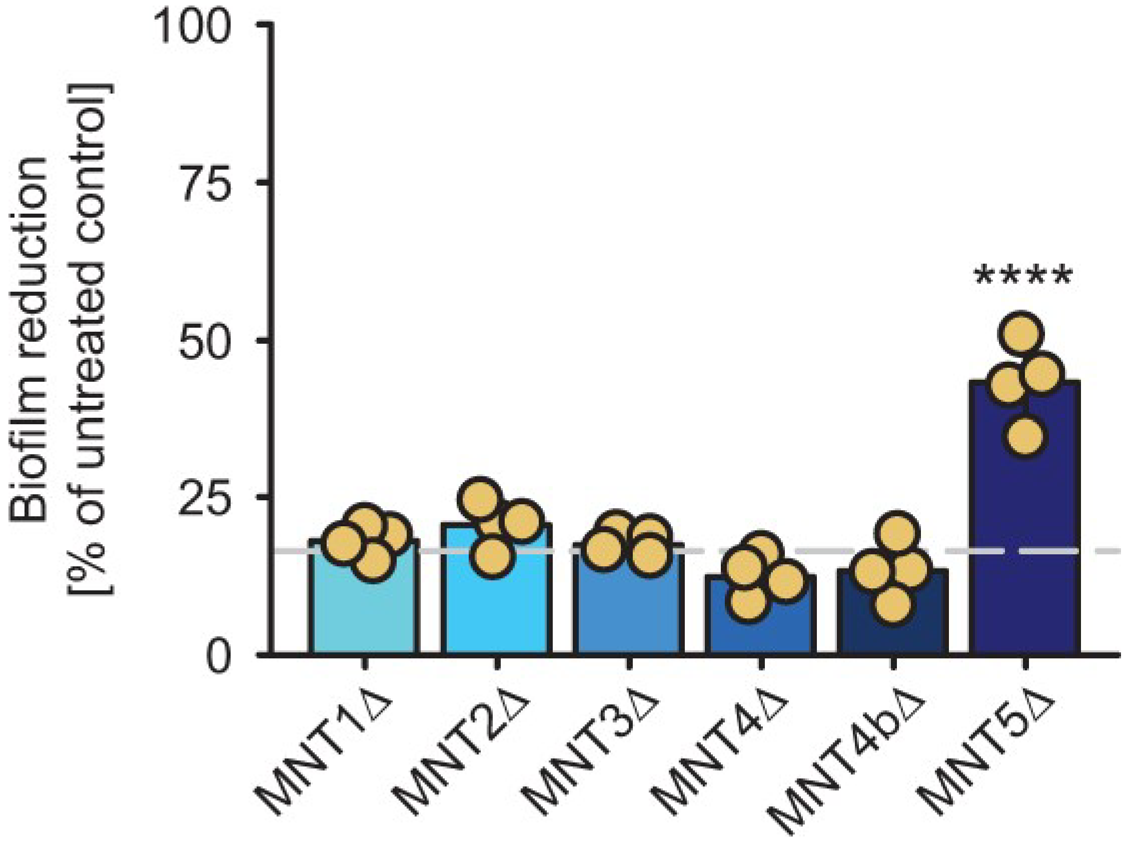

Fig S2 Neutrophil engagement of wild-type and *mnt1*Δ*, mnt2*Δ*, mnt3*Δ*, mnt4*Δ*, mnt4b*Δ*,* and *mnt5*Δ*.* Three independent biological replicates, each with 12 technical replicates (cell events), were performed. Error bars represent standard deviation. A non-parametric Kruskal–Wallis one-way analysis of variance with an uncorrected Dunn's multiple comparison test was used, with a significant *p*-value indicated as <0.0001.

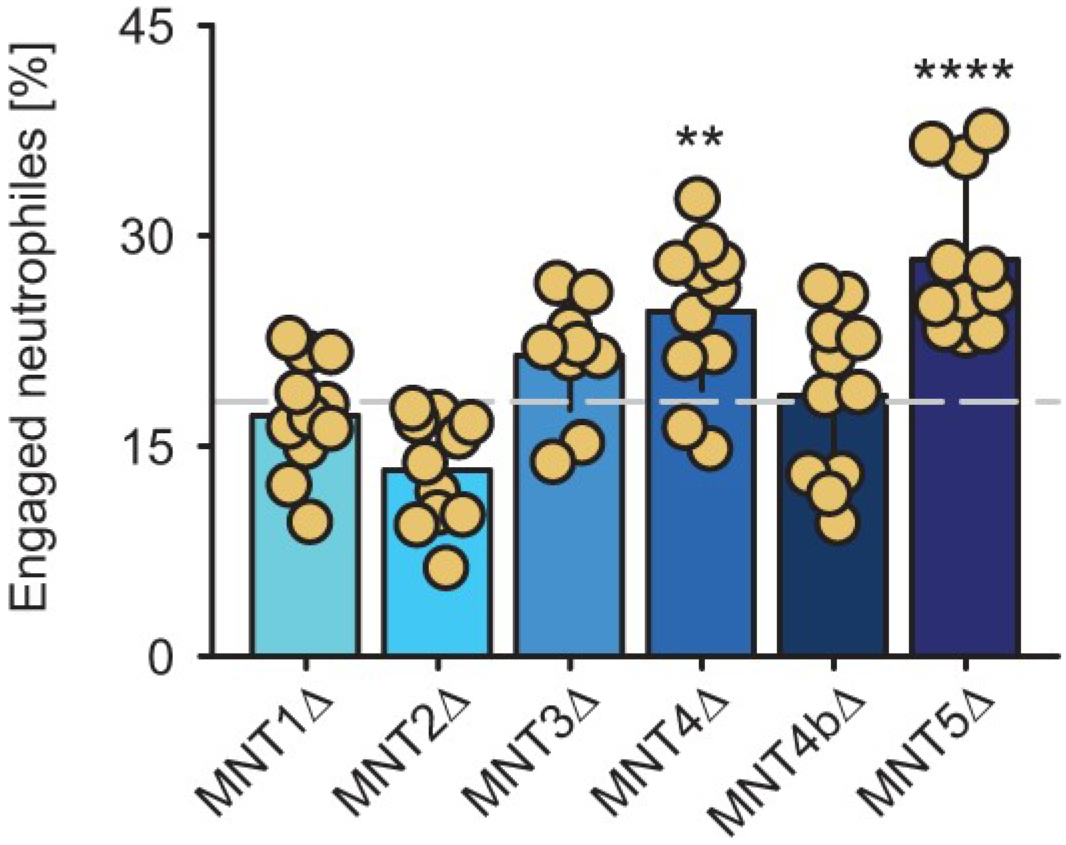

References

1. Lockhart, S. R. *et al.* Simultaneous Emergence of Multidrug-Resistant Candida auris on 3 Continents Confirmed by Whole-Genome Sequencing and Epidemiological Analyses. *Clin Infect Dis* **64**, 134-140, doi:10.1093/cid/ciw691 (2017).
2. Zarnowski, R. *et al.* A common vesicle proteome drives fungal biofilm development. *Proc Natl Acad Sci U S A* **119**, e2211424119, doi:10.1073/pnas.2211424119 (2022).
3. Horton, R. M., Hunt, H. D., Ho, S. N., Pullen, J. K. & Pease, L. R. Engineering hybrid genes without the use of restriction enzymes: gene splicing by overlap extension. *Gene* **77**, 61-68, doi:10.1016/0378-1119(89)90359-4 (1989).
4. Zarnowski, R., Sanchez, H. & Andes, D. R. Large-scale production and isolation of Candida biofilm extracellular matrix. *Nat Protoc* **11**, 2320-2327, doi:10.1038/nprot.2016.132 (2016).
5. Horton, M. V. *et al.* Candida auris Cell Wall Mannosylation Contributes to Neutrophil Evasion through Pathways Divergent from Candida albicans and Candida glabrata. *mSphere* **6**, e0040621, doi:10.1128/mSphere.00406-21 (2021).
6. Nett, J. E., Cain, M. T., Crawford, K. & Andes, D. R. Optimizing a Candida biofilm microtiter plate model for measurement of antifungal susceptibility by tetrazolium salt assay. *J Clin Microbiol* **49**, 1426-1433, doi:10.1128/JCM.02273-10 (2011).
7. Johnson, C. J., Davis, J. M., Huttenlocher, A., Kernien, J. F. & Nett, J. E. Emerging Fungal Pathogen Candida auris Evades Neutrophil Attack. *mBio* **9**, doi:10.1128/mBio.01403-18 (2018).
8. Nett, J. *et al.* Putative role of beta-1,3 glucans in Candida albicans biofilm resistance. *Antimicrob Agents Chemother* **51**, 510-520, doi:10.1128/AAC.01056-06 (2007).
9. Graus, M. S. *et al.* Mannan Molecular Substructures Control Nanoscale Glucan Exposure in Candida. *Cell Rep* **24**, 2432-2442 e2435, doi:10.1016/j.celrep.2018.07.088 (2018).
10. Shibata, N., Suzuki, A., Kobayashi, H. & Okawa, Y. Chemical structure of the cell-wall mannan of Candida albicans serotype A and its difference in yeast and hyphal forms. *Biochem J* **404**, 365-372, doi:10.1042/BJ20070081 (2007).
11. Hobley, P., Howarth, O. & Ibbett, R. N. 1H and 13C NMR Shifts for Aldopyranose and Aldofuranose Monosaccharides: Conformational Analysis and Solvent Dependence. *Magnetic Resonance in Chemistry* **34**, 755-760, doi:https://doi.org/10.1002/(SICI)1097-458X(199610)34:10<755::AID-OMR950>3.0.CO;2-U (1996).
12. Lowman, D. W. *et al.* Mannan structural complexity is decreased when Candida albicans is cultivated in blood or serum at physiological temperature. *Carbohydr Res* **346**, 2752-2759, doi:10.1016/j.carres.2011.09.029 (2011).
13. Bastos, R. *et al.* Covalent connectivity of glycogen in brewer's spent yeast cell walls revealed by enzymatic approaches and dynamic nuclear polarization NMR. *Carbohydr Polym* **324**, 121475, doi:10.1016/j.carbpol.2023.121475 (2024).

14 Lowman, D. W. *et al*. Glucan and glycogen exist as a covalently linked macromolecular complex in the cell wall of Candida albicans and other Candida species. *Cell surface (Amsterdam, Netherlands)* **7**, 100061, doi:10.1016/j.tcsw.2021.100061 (2021).

15 Zang, L. H., Howseman, A. M. & Shulman, R. G. Assignment of the 1H chemical shifts of glycogen. *Carbohydr Res* **220**, 1-9, doi:10.1016/0008-6215(91)80001-4 (1991).

16 Zhang, X., Leemhuis, H. & van der Maarel, M. Characterization of the GH13 and GH57 glycogen branching enzymes from Petrotoga mobilis SJ95 and potential role in glycogen biosynthesis. *PLoS One* **14**, e0219844, doi:10.1371/journal.pone.0219844 (2019).

17 Zhu, Y., Delbianco, M. & Seeberger, P. H. Automated Assembly of Starch and Glycogen Polysaccharides. *J Am Chem Soc* **143**, 9758-9768, doi:10.1021/jacs.1c02188 (2021).

18 Aimanianda, V. *et al*. The Dual Activity Responsible for the Elongation and Branching of beta-(1,3)-Glucan in the Fungal Cell Wall. *mBio* **8**, doi:10.1128/mBio.00619-17 (2017).

19 Xin, Y. *et al*. Immune-enhancing effect of water-soluble beta-glucan derived from enzymatic hydrolysis of yeast glucan. *Biochem Biophys Rep* **30**, 101256, doi:10.1016/j.bbrep.2022.101256 (2022).
